# An open source convolutional neural network to detect and localize distal radius fractures on plain radiographs

**DOI:** 10.1007/s00068-024-02731-4

**Published:** 2025-01-17

**Authors:** Koen D. Oude Nijhuis, Britt Barvelink, Jasper Prijs, Yang Zhao, Zhibin Liao, Ruurd L. Jaarsma, Frank F. A. IJpma, Joost W. Colaris, Job N. Doornberg, Mathieu M. E. Wijffels

**Affiliations:** 1 Department of Orthopaedic Surgery, University Medical Centre Groningen and Groningen University, Hanzeplein 1, 9713PZ Groningen, the Netherlands; 2 Department of Trauma Surgery, University Medical Centre Groningen and Groningen University, Hanzeplein 1, 9713PZ Groningen, the Netherlands; 3 Department of Orthopaedics and Sports Medicine, Erasmus University Medical Centre, Rotterdam, The Netherlands; 4 Department of Orthopaedic Surgery, Flinders University and Medical Centre, Adelaide, South Australia Australia; 5 Australian Institute for Machine Learning, Adelaide, Australia; 6 Trauma Research Unit, Department of Surgery, Erasmus MC, University Medical Centre Rotterdam, Rotterdam, the Netherlands

**Keywords:** Trauma, Distal radius fractures, Wrist, Artificial intelligence

## Abstract

**Purpose:**

Distal radius fractures (DRFs) are often initially assessed by junior doctors under time constraints, with limited supervision, risking significant consequences if missed. Convolutional Neural Networks (CNNs) can aid in diagnosing fractures. This study aims to internally and externally validate an open source algorithm for the detection and localization of DRFs.

**Methods:**

Patients from a level 1 trauma center from Adelaide, Australia that presented between 2016 and 2020 with wrist trauma were retrospectively included. Radiographs were reviewed confirming the presence or absence of a fracture, as well as annotating radius, ulna, and fracture location. An internal validation dataset from the same hospital was created. An external validation set was created with two other level 1 trauma centers, from Groningen and Rotterdam, the Netherlands. Three surgeons reviewed both sets for DRFs.

**Results:**

The algorithm was trained on 659 radiographs. The internal validation set included 190 patients, showing an accuracy of 87% and an AUC of 0.93 for DRF detection. The external validation set consisted of 188 patients, with an accuracy and AUC were 82% and 0.88 respectively. Radial and ulnar bone segmentation on the internal validation was excellent with an AP50 of 99 and 98, but moderate for fracture segmentation with an AP50 of 29. For external validation the AP50 was 92, 89 and 25 for radius, ulna, and fracture respectively.

**Conclusion:**

This open-source algorithm effectively detects DRFs with high accuracy and localizes them with moderate accuracy. It can assist clinicians in diagnosing suspected DRFs and is the first radiograph-based CNN externally validated on patients from multiple hospitals.

## Introduction

Distal radius fractures (DRFs) are among the most common fractures seen in emergency departments, frequently occurring after a fall onto the outstretched hand [1]. Due to the urgency to make early management decisions in the emergency departments, a tentative assessment of the radiographs is often made by a junior, non-radiology trained, clinician before the definitive radiology report is available [2]. Additionally, there might be a threshold to consult a supervisor 24/7 a day. Interpretational errors can have significant consequences for the patient, including delayed treatment and consequently poorer outcomes [3, 4].

In the field of Artificial intelligence (AI) coined Computer Vision, Convolutional Neural Networks (CNN) have been of particular interest as a possible aid to (non-radiology trained) clinicians because of their ability to ‘read’ images, allowing them to recognize fractures [5]. Deep Learning, a form of machine learning based on these artificial neural networks, has garnered significant attention to achieve this goal. CNNs have exhibited superior capacity to radiologists in detecting DRFs on plain radiographs in recent studies with sensitivity and specificity between 81–94% and 78–100%, respectively (see Table 1) [6–11]. One CNN, created by Imagen, has been approved by the United States Food and Drug Administration (FDA) and is commercially available, after a study demonstrated that emergency department clinicians show significant improvement with aid of this software [9, 12]. However, this algorithm is not freely accessible for testing which limits its broad application in clinical practice and prevents further external validation in its current format.

**Table 1:** Previous studies investigating DRF detection on plain radiographs

Year	Author	Dataset size	CNN Performance	Conclusion
2017	Kim et al	1389	Sensitivity: 90% Specificity: 88% AUC: 0.95	Comparable to state-of-the-art fracture detection models
2018	Lindsey et al	34,990	Sensitivity: 94% Specificity: 95% AUC: 0.99	Accident & Emergency clinicians performed significantly better when aided by AI
2019	Thian et al	7356	Sensitivity: 98% Specificity: 73% AUC: 0.90	CNN has high sensitivity in terms of detection and localization of the fracture
2019	Gan et al	2340	Sensitivity: 90% Specificity: 96% AUC:0.96	Similar performance to orthopaedic surgeons Superior performance to radiologists
2020	Blütghen et al	824	Sensitivity: 80–90% Specificity: 78%−100% AUC: 0.87–0.95	Performance similar to that of radiologists

The clinical application of the algorithms for orthopaedic trauma is still limited because of limited availability of open source algorithms that have been validated both internally and externally [13]. Most available studies have primarily trained and tested their algorithms on internal data sets, likely resulting in bias of CNN performance (i.e., overfitting) that cannot be replicated by using an external validation dataset. For example, Blüthgen et al. tested their algorithm with both internal validation and external validation datasets [6]. Their algorithm showed significantly poorer performance on the external radiographs, demonstrating the importance of incorporating external data in CNN training. As such, the philosophy of the current study is to present a trained open source algorithm that has been internally and externally validated. Furthermore, we will allow other centers to extend the validation of this algorithm for free and train it further to optimize its performances.

The aim of this study is to validate the performance of a recently developed ‘open source’ CNN with the ability to detect and localize DRFs in postero-anterior (PA) and lateral radiographs. Diagnostic accuracy, sensitivity, specificity, and area under the receiving operating characteristic curve (AUC) of this algorithm are the main outcome parameters.

## Patients/methods

### Ethical approval & guidelines

Ethical approval was granted by the ethics committee (CALHN 13991). There are no conflicts of interest. The study was performed in accordance with the Clinical AI Research (CAIR) checklist, a guideline for AI research in a healthcare setting [14].

### Training data set

Patients with a suspected DRF presenting to the emergency department of a level-1 trauma center (hospital 1) after sustaining a wrist trauma were retrospectively included from the years 2016 to 2020, when both PA, lateral radiographs were present. The patient files were identified in the picture archiving and communication system (PACS) using ICD-9 diagnostic codes, i.e., “fracture” and “radius”, and afterwards manually checked if they were indeed a wrist radiograph. In the case of a wrist radiograph, they were checked against the exclusion criteria: pathology other than DRF (not including concomitant ulnar styloid fractures), presence of epiphyseal growth plates, poor image quality (e.g., artefacts, noise, under- or overexposure and casts that severely decrease image quality; such that physicians also cannot read the radiograph) and objects obstructing the distal radius. All included wrist radiographs were then assessed for a DRF was present or not.

### Convolutional neural network

Modelled on the human visual cortex, CNNs learn and acquire knowledge through neural pathways consisting of various layers, including an input layer, hidden layer(s), and an output layer. Complex mathematical operations are performed between the nodes and their weighted connections, ultimately resulting in algorithm training. In order to save substantial time and computational power, it is worthwhile using an established CNN that has already been trained to identify features in images. We used the open source CNN ImageNet, which has previously been trained with more than one million nonmedical images with over 1,000 object categories [15]. The performance of this model with our radiographs was evaluated in terms of accuracy, sensitivity, specificity, and AUC. The CNN was programmed in Python Version 3.6.8 along with Scikit-learn (0.20.3) and TensorFlow (1.13.1).

**Fig. 1:**
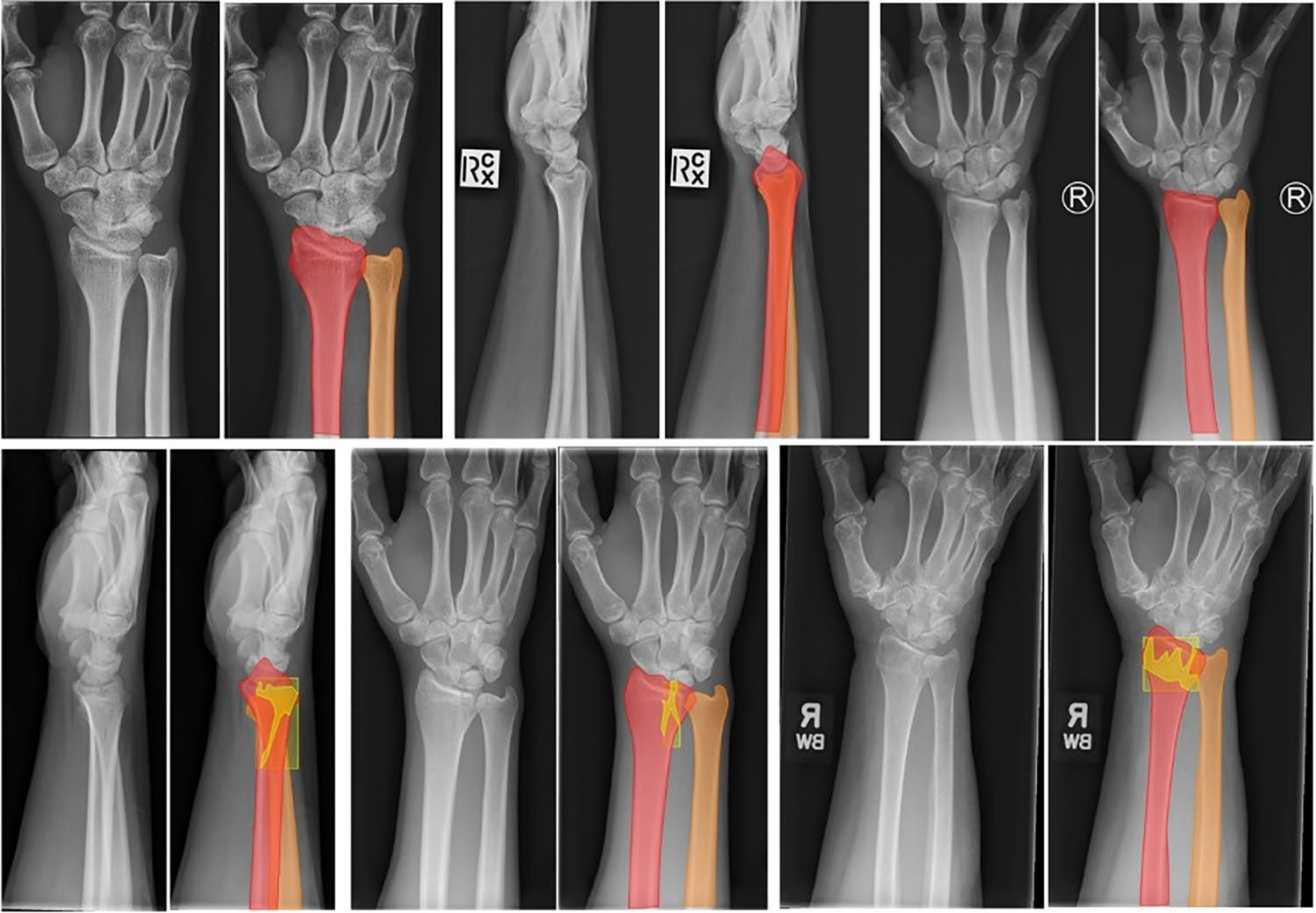
The upper row of images demonstrates, from left to right, the annotations of ulna and radius in non-fracture radiographs in oblique, lateral and PA view. The lower row of images shows the annotations of ulna, radius, and the region of interest (e.g., fracture site) using both a rectangular and polygon tool

The wrist radiographs were exported from PACS as Digital Imaging and Communications in Medicine (DICOM) files and subsequently anonymized with free open source software DICOM Cleaner [PixelMed Publishing, LLC]. The anonymized files were uploaded to an online computer vision training data platform LabelBox [16]. On LabelBox, two independent reviewers each confirmed the presence or absence of a fracture, and then annotated the radius, ulna, and region of interest (ROI) of the fracture, if present, on the training dataset. The reviewers had access to the original radiologist report, and support from an experienced orthopaedic trauma surgeon in case of doubt. They did not have access to subsequent follow-up radiographs. As demonstrated in Fig. 1, the fracture ROI was selected with both a rectangle (bounding box) and a polygon tool (segmentation) encompassing the entire fracture area. All labels and annotations of the initial training dataset were evaluated by a senior reviewer (KDON and JP). The consensus agreement between the two initial reviewers and the senior reviewers formed the ground truth for the training set.

The dataset was then sent to an institute specialized in Machine Learning to train the CNN model. The deep learning model that was evaluated in this study was a Mask R-CNN based on Detectron2 (an image detection and segmentation algorithm), the backbone of which is a ResNet-50 and an RPN module [17]. Specifically, our network is trained with stochastic gradient descent (SGD) for 6250 iterations with the initial learning rate being 0.02 and a mini-batch of 8 images, changing models parameters till the optimum values have been reached. The learning rate, allowing the training to become more precise with each iteration, is reduced by a factor of 10 at iteration 3750 and 5625, respectively. Weight decay and momentum, each a way to create a complex model without overfitting, are set as 0.0001 and 0.9, respectively. Each image has gone through augmentation in the training process, by rotating, flipping, and zooming in. We initialize our backbone networks with the weights pre-trained on ImageNet, allowing the algorithm to be familiar with images detection before specifying the training to DRFs. All experiments were all performed with PyTorch framework on an Nvidia V100 GPU.

The code has been made publicly available for further training or external validation on GitHub here: https://github.com/AIML-MED/DRF_Classification_Public.

### Internal validation

Subsequently an internal validation was performed to test the algorithm. Patients from hospital 1 were collected. Three surgeons (two trauma surgeons, one orthopaedic surgeon: FFAIJ, MMEW, JWC) were independently shown all radiographs and reviewed these for the presence or absence of a DRF and came to a consensus. They did not have access to the original radiology report, or any follow-up images when reviewing. This was considered the ground truth of the internal validation. Just like on the training set, two independent reviewers (HVL, OC) then annotated the radius, ulna, and ROI of the fracture (if present) and this was evaluated by a senior reviewer (KDON).

### External validation

To properly test the generalizability of the algorithm, an external validation was performed. Patients from two different level 1 trauma centers (hospital 2 and 3) were collected. The same three surgeons independently reviewed these cases for the presence or absence of a DRF and came to a consensus. This was considered the ground truth of the external validation. The radiographs were then annotated, identical to the process described in the internal validation.

### Statistics

Continuous variables are expressed as the mean with its standard deviations, and categorical variables as percentages or frequencies. Accuracy describes the percentage of correctly identified fractures. Sensitivity is the proportion of correctly identified fractures out of all fractures. Specificity is the proportion of correctly identified non-fractures out of all non-fractures. Positive predictive value (PPV) is the ratio of patients truly diagnosed as positive to all those who had positive test results. Negative predictive value (NPV) is the ratio of subjects truly diagnosed as negative to all those who had negative test results. The AUC denotes the likelihood of a binary classifier system to correctly separate a particular variable into either a zero or one. AUC of 1 indicates perfect separation performance, whereas 0.5 indicates no ability to separate better than chance.

We also use detection metrics Average Precision 50% Overlap (AP50) to evaluate our model. AP50 is a commonly used metric to evaluate object detection models. An overlap between the manual localization and performance of the algorithm of at least 50% is considered correct, below 50% incorrect. The higher AP50 is, the better the model is in localizing objects. The AP50 is given for a bounding box, a less precise method of localization using a rectangle, and for segmentation, a precise method of localization using a polygon. See Fig. 1 for examples of manual segmentation and bounding boxes. Results are given as an average over all bounding box localizations and segmentations, and for each individual group: radius, ulna, and fracture. If a fracture is completely missed, this is also counted as incorrect.

## Results

### Demographics

A total of 6544 radiographs taken between 2016 and 2020 were extracted from the record system of hospital 1. Due to the anonymization and randomization of the training set, it is not possible to ascertain patient numbers or characteristics. After eliminating images subject to our exclusion criteria, 659 radiographs (PA, lateral or oblique) were available for further analysis. According to the ground truth, 315 DRFs were diagnosed and 344 had no visible fractures. All these radiographs were annotated and used to train the algorithm.

The internal validation consisted of 190 patients, 145 patients with a DRF and 45 without a fracture, consisting in total of 498 radiographs (PA and lateral, and oblique when present). The external validation consisted of 188 patients, 134 patients with a DRFs and 54 without a fracture, consisting in total of 376 radiographs (PA and lateral). The internal validation and external validation numbers are based on comparative studies on the matter [7, 8, 11].

### CNN performance: internal validation

The accuracy of the algorithm in detecting DRFs on the internal validation was 87%. The sensitivity of the CNN for recognizing a DRF was 85%, the specificity was 96%. The PPV was 98% and the NPV was 66%. The AUC of the algorithm for accurately predicting the presence of a DRF was 0.93.

For localization results in internal validation set using a bounding box, our model achieves an average AP50 of 83 over all entities. For the localization of the radial bone bounding box the AP50 scored 99, the ulnar bone scored 100 and for fracture localization it scored 50. For segmentation using a polygon in the internal validation set, our model achieves an average AP50 of 75. For the segmentation of the radial bone the AP50 scored 99, the ulnar bone scored 98 and for fracture localization it scored 29.

### CNN performance: external validation

The accuracy of the algorithm in detecting DRFs on the external validation was 84%. The sensitivity of the CNN for recognizing a DRF was 82% while the specificity was 89% The PPV was 95% and the NPV was 67%. The AUC of the algorithm for accurately predicting the presence of a DRF was 0.88.

For localization results in external validation set using a bounding box, our model achieves an average AP50 of 80 over all entities. For the localization of the radial bone bounding box the AP50 scored 95, the ulnar bone scored 93 and for fracture localization it scored 52. For segmentation using a polygon in the external validation set, our model achieves an average AP50 of 69. For the segmentation of the radial bone the AP50 scored 92, the ulnar bone scored 89 and for fracture localization it scored 25.

## Discussion

The use of AI may improve accuracy of fracture detection on radiographs of patients at risk for a DRF. Studies exploring AI-based fracture recognition on radiographs have produced promising results with performance similar or superior to that of radiologists and orthopaedic surgeons (Table 1). Possible reasons why algorithms surpass clinicians are various; algorithms do not get tired, they are trained to do one specific task, and might recognize subtleties missed by humans [8]. Clinical usability of algorithms, however, is limited due to poor external validation, of promising algorithms so far [13]. External validation of an algorithm is needed to correct for a false accuracy due, amongst others, significant image pre-processing.

This study has some limitations. First of all, all images of poor quality were excluded, defined as those with artefacts, noise or under- or overexposed, and other fractures, introducing a selection bias and possibly leading to a form of overfitting. External validation in a less processed dataset might overcome this limitation. Secondly, the consensus between the two independent reviewers and the senior reviewers was considered the ground truth for the training set. Their annotations may be prone to mistakes and subject to a learning curve throughout the process. This could influence the training of the algorithm. Further research is needed to explore if adding patient characteristics to the algorithm results in improved detection. Also, oblique radiographs are regularly used in Australia, but not in the Netherlands. This explains the difference in number of radiographs between the internal validation and external validation, despite having a similar number of patients. This could further explain the difference between the internal validation and external validation results. Finally, physicians look at the different radiographic views at the same time, allowing them to make a judgement based on multiple radiographs. The algorithm is only capable of looking at one view at the same time, and the accuracy is calculated as an average of these views. Combining the views might positively influence the algorithm and might be worth exploring in the future.

This study showed that the open source algorithm, that was trained with 659 radiographs, has an accuracy of 87%, a sensitivity of 85%, a specificity of 96%, a PPV of 98%, a NPV of 66% and an AUC of 0.93 for detecting distal radius fractures. The external validation had an accuracy of 84%, sensitivity of 82%, specificity of 89%, a PPV of 95%, a NPV of 67% and AUC of 0.88.

Localization using a bounding box was excellent, with an AP50 of 99.0 and 100 for the radius and ulna respectively, and good for fracture localization with an AP50 of 50.0. The external validation showed similar results, with an AP50 of 94.8, 92.8 and 51.8 for the radius, ulna, and fracture respectively.

Segmentation of the radius and ulna is excellent, with an AP50 of 98.9 and 97.6 respectively, but moderate for fracture segmentation with an AP50 of 29.3. The external validation showed slightly worse results, with an AP50 of 92.4, 88.6 and 24.8 for the radius, ulna, and fracture respectively.

To date, this is the first CNN fracture detection tool that has been externally validated using radiographs from more than one hospital. The algorithm showed a very high PPV on external validation, implying usefulness in clinical practice as a tool to diagnose a DRF is promising. The sensitivity was higher than the specificity, indicating that adding more DRF cases might further improve results. However, it was chosen to use all available DRFs in the trainings set, rather than remove patients and aim for a 50/50 split between DRF and no-fracture cases, explaining the small disbalance in the training set. Further research has to be done regarding its clinical applicability, since previous studies have shown clinicians’ accuracy may improve using fracture detection algorithms [18].

**Fig. 2:**
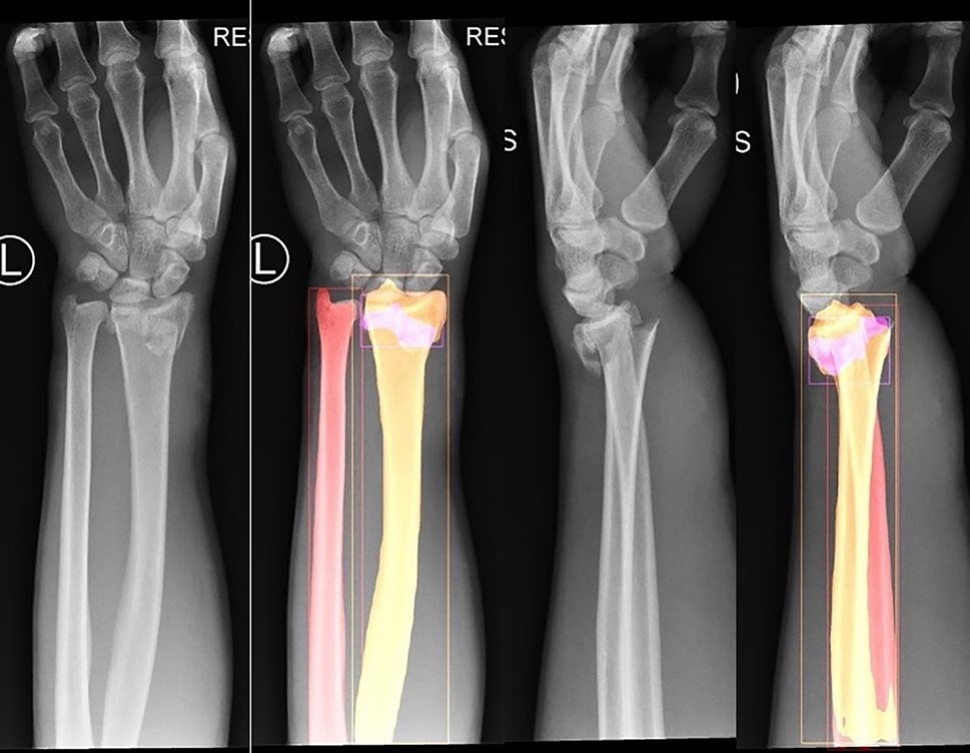
AP and lateral view of a patient with a DRF in the internal validation set. The algorithm localizes the radius (orange), ulna (red) and fracture (purple)

**Fig. 3:**
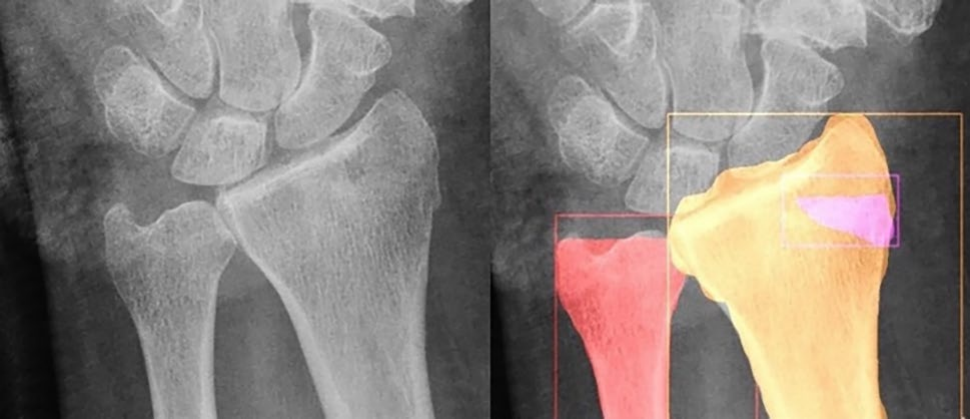
A zoomed in view of the output of the algorithm, on a subtle fracture, obstructed by a cast

**Fig. 4:**
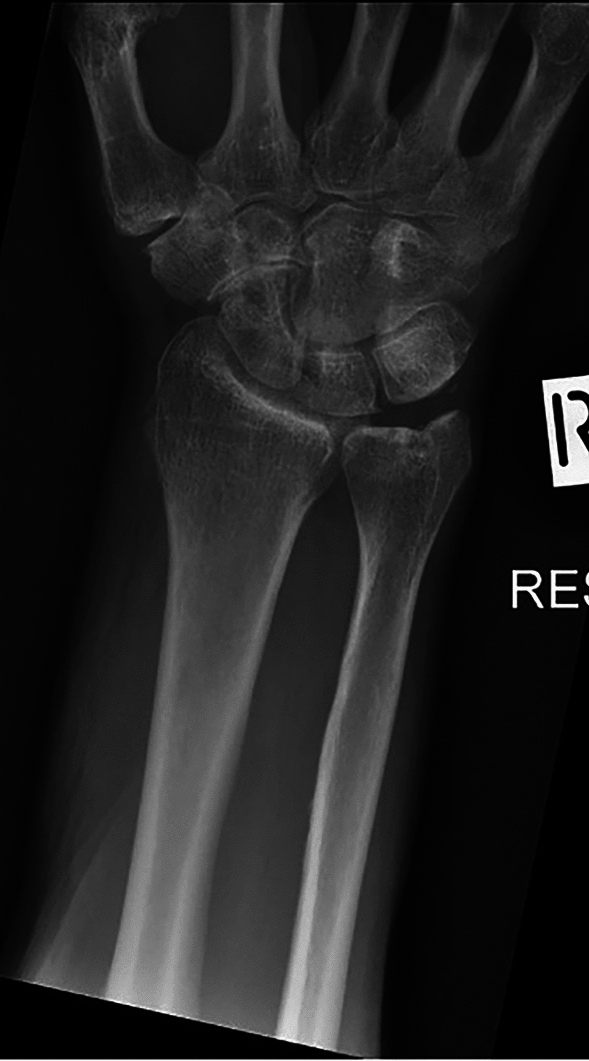
Patient from the internal validation set. The algorithm missed this fracture

Not only is the algorithm able to indicate whether there is a DRF or not, it is also capable of localizing it (see Fig. 2). This makes it easier for the physician to use the algorithm as a diagnostic tool when in doubt whether there is a fracture or not (Fig. 3). It also localizes and segments the radial and ulnar bone, even on lateral radiographs. Perhaps not necessarily useful in a clinical setting as of yet, but future research could benefit from having automated bones detection and annotation by an algorithm, more quickly than a human can, for instance in pre-operative planning. Examination of the output of the algorithm shows that the algorithm struggles the most with diagnosing subtle, non-displaced intra-articular fractures (see Fig. 4). The results for fracture segmentation are moderate, partially explained by the fact that if a fracture is missed or incorrectly predicted to be there, the AP50 drastically decreases. Perhaps more training data can improve these results. The results for localization and segmentation of the radius and ulna are almost perfect.

Compared to other studies that created CNNs for detecting DRFs, our algorithm performed with similar sensitivity and specificity. Our AUC of 0,93 was similar to other studies on their internal validation. While we did not test our algorithm versus radiologists or surgeons, the physicians in previous studies performed similar to our algorithm. Future studies should focus on differences in performance of physicians with or without the aid of an algorithm in detection of fractures, to strengthen the already published data. Lindsey et al. showed a 47% reduction of missed fractures in clinicians using their algorithm, with their sensitivity improving from 81 to 92% [9]. Furthermore, the current dataset should be increased, to see if a higher accuracy can be obtained. Also, more complex fracture segmentation techniques should be tried to see if results can be improved. Perhaps the algorithm can get a more complete idea of the fracture when trained on several radiograph views at the same time, as well as 3D-imaging techniques such as computed tomography (CT) scans.

The created algorithm will be made freely available to the public, allowing other researchers to further improve and test the algorithm. This will provide more information of the practical applications of this algorithm on the one hand, and insight if larger patient numbers from other hospitals will increase the accuracy on the other hand. No data is available on this so far.

In conclusion, the algorithm has demonstrated high accuracy in detecting DRFs on radiographs and moderate accuracy in localizing them. This study shows similar results to previous algorithms, however the extensive external validation suggests clinical useability. Future studies are needed to compare this model’s performance (in addition) to that of human observers. Other centers will be able to use this algorithm, either by further training it, or by performing an external validation themselves.

## Data Availability

No datasets were generated or analysed during the current study.
